# Synthesis of Au@polymer nanohybrids with transited core-shell morphology from concentric to eccentric Emoji-N or Janus nanoparticles

**DOI:** 10.1038/s41598-018-24078-8

**Published:** 2018-04-10

**Authors:** Nekane Guarrotxena, Olga García, Isabel Quijada-Garrido

**Affiliations:** Instituto de Ciencia y Tecnología de Polímeros, Consejo Superior de Investigaciones Científicas (ICTP-CSIC), c/Juan de la Cierva, 3 E-28006 Madrid, Spain

## Abstract

The combination of multifunctionality and synergestic effect displayed by hybrid nanoparticles (NPs) has been revealed as an effective stratagem in the development of advanced nanostructures with unique biotechnology and optoelectronic applications. Although important work has been devoted, the demand of facile, versatile and efficient synthetic approach remains still challenging. Herein, we report a feasible and innovative way for polymer-shell assembling onto gold nanoparticles in competitive conditions of hydrophobic/hydrophilic feature and interfacial energy of components to generate core-shell nanohybrids with singular morphologies. The fine control of reaction parameters allows a modulated transformation from concentric to eccentric nanostructure-geometries. In this regard, a rational selection of the components and solvent ratio guarantee the reproducibility and efficiency on hybrid-nanoassembly. Furthermore, the simplicity of the synthetic approach offers the possibility to obtain asymmetric Janus NPs and new morphologies (quizzical-aspheric polymer-shell, named Emoji-N-hybrids) with adjustable surface-coating, leading to new properties and applications that are unavailable to their symmetrical or single components.

## Introduction

The outstanding synthetic progress made in achieving different patterns of single-component nanostructures, crystal (sphere, cube, wire, and plate)^1,2^ or polymer (sphere, toroid, vesicle)^3,4^, respectively has not yet been matched in the more complex (multi)functional (nano-objetcs). Recently, the knack of multifunctionality and synergistic effects of hybrid nanostructures, involving at least two different component domains, has arisen as an effective approach in the quest for new structures^5–8^. Indeed, exploring the intersection between the domains can offer synthetic pathways to attain structures, far away from the conventional single-component systems with engineered properties and desired functionalities. Thus far, core-shell nanostructures, through their composition, morphology and interfacial energy tunability, are the elementary motif to provide a rational synthesis and design of multicomponent nanohybrids^9,10^. Their key-implication in high technological applications as targeted drug delivery, antimicrobial therapy, sensors, nanoactuators, electronic and catalytic devices does reinforce the growing demand of innovative, precise, and practicable synthesis protocols^11–15^.

Most of the current methods, however, keep on the multiple-step paradigm, frequently counting on interfacial adhesion and modification of pre-built components. A case to this point is our recent fabrication of uniform and spherical plasmonic Au-polymer core-shell nanohybrids^7^. Notwithstanding the successful yield of single fully-polymer coated AuNPs; a previous synthesis of the thiolated copolymer stabilizing-layer followed by two-steps, such as ligand exchange of initial citrate NPs capping and subsequent crosslinking polymerization of MEO_2_MA around the copolymer coated NPs, could still result in somehow tedious for the core-shell generation. Other approaches, all involving AuNPs as core-seeds^16–23^; in spite of their success toward more complex structures, similarly suffer from a limited simplicity, low throughput and low rentability/sustainability of production. These considerations fuel the demand for alternative synthetic methods. Although, several researchers have reported remarkable studies on one-pot syntheses of anisotropic Au-polymer hybrid nanoparticles^24,25^; they are restricted to Janus NP morphologies and still a versatile synthetic strategy capable of creating metal@polymer nanohybrids, with fine-tuned structure (size, shape, morphology) and composition; and with defined optical properties in a controlled way is highly desirable.

**Figure 1:**
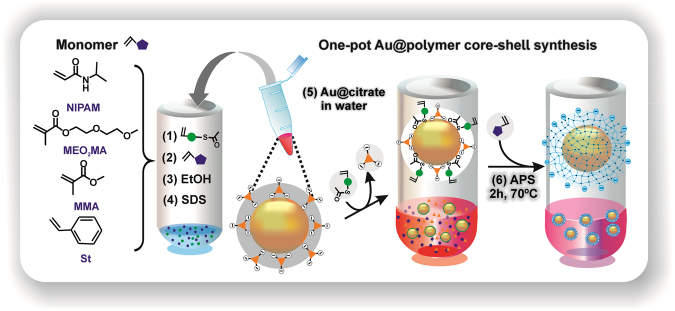
Cartoon describing: (right) Chemical route to ¨one-pot¨ synthesizing core-shell Au@polymer nanohybrids and (left) Structures of monomers used for polymer-shell coverage of AuNP.

Here we report on a one-pot synthesis of singular nanohybrid species with tunable anisotropic polymer coverage via interfacial energy and surface ¨wetting/dewetting¨ adjustment of the components in aqueous media. Interestingly, while the use of methacrylate-monomer tagged-AuNPs, as seeding system, for self-assembly of diverse selected acrylic/vinyl-monomers by precipitation-polymerization (Fig. 1) generates, in one-step, Au@polymer hybrid NPs with a wide sort of polymer shells; the fine-tuning of ethanol-water proportion efficiently yields morphologies ranging from core-shell-like to eccentric or Janus structures. Furthermore, this simple modification of usual precipitation-polymerization reaction overcomes difficulties, inherent to time-lapse control for Au-seeds addition^16,17^, absence of shell-thickness tunability^18^, mixed bimodal patterned NP distribution^19^, and complexness^22,23^, reported in previous synthetic approaches. Even when the conceptual use of interfacial energy for nanohybrid syntheses with tunable morphology is not new; the reported works used enriched isopropanol media and are only applicable to highly hydrophilic polymers as polyacrylic acid (PAA) with suitable functional groups to interact with NPs surface^26,27^. Additionally, our method provides a controlled composition domain, since it is likely to be applicable to a large number of polymers.

## Experimental

### Materials

The monomers, 2-(2-methoxyethoxy)ethyl methacrylate (MEO_2_MA, Aldrich 95%) was purified by passing through a neutral alumina column; *N*-isopropylacrylamide (NIPAM) was purified by recrystallization, from a hexane/toluene mixture (90/10 vol. %); Styrene (St, Merck > 99%) and methyl methacrylate (MMA, Merck > 99%) were used as received. For the synthesis of 2-(2-(2-(acetylthio)ethoxy)ethoxyl)ethyl methacrylate (AcSEO_2_MA), 2-(2-(2-chloroethoxy)ethoxy)ethan-1-ol (96%, Aldrich), methacryloyl chloride (97%, Aldrich), triethylamine (99%, Scharlau), dichlorometane (DCM) (99%, Aldrich), anhydrous sodium sulfate (99%, Qemical), potassium iodide (99.5%, Panreac), potassium thioacetate (98%, Aldrich) and acetonitrile (99.9%, Scharlau) were employed as received. All chemical reagents: ammonium persulfate (APS) (>98%, Fluka); tetraethylene glycol dimethacrylate (TEGDMA) (>90%, Fluka); sodium dodecyl sulfate (SDS) (Fluka); tetrachloroauric (III) acid (HAuCl_4_·3H_2_O) (≥99.9%, Aldrich) and trisodium citrate dihydrate (Sigma-Aldrich)] were used as received. Milli-Q water was used in all experiments. Solvents were dried by standard methods or by elution through a Pure Solv Innovative Technology column drying system.

### Synthesis of AcSEO_2_MA

The acetylthiolated methacrylic monomer was synthesized according the two-step protocol previously reported^28^ with slight modifications (Supplementary Information).

### Synthesis of Gold Nanoparticles (AuNPs)

Uniform colloidal AuNPs (58 ± 6 nm) were prepared by a seeded mediated growth method derived from the citrate-reduction method by using 15 nm gold seeds^7,29^. Briefly, 0.5 mL of sodium citrate (1% wt), 0.5 mL HAuCl_4_ (25 mM) and 0.1 mL of NaOH (0.165 M) solution were added to the seeds solution, previously diluted with the same volume of water, and allowed to reflux for 20 min. This addition cycle was sequentially repeated twice, using double volume of all reactants, followed by a series of additional cycles without the addition of water. The solution was then allowed to cool to room temperature, and refrigerated until further use.

### Synthesis of Au@Polymer Core-shell Hybrids

The Au@polymer nanohybrids were synthesized through heterogeneous polymerization in water. Previously to the polymerization step, 1 mL of the as-synthesized AuNPs was centrifugated (4500 rpm, 30 min.) to remove the free citrate ligand. A typical procedure for the shell synthesis is described below for sample Au@pMEO_2_MA-G1 (Table 1). In a tube equipped with a stirrer and a N_2_ gas inlet, reactive were added in the following order: 4.75 μL of a solution of AcSEO_2_MA monomer in ethanol (1.72 10^−3^ mol), MEO_2_MA monomer (6.5 10^−2^ mmol), 34 μL of a solution of TEGDMA crosslinker (1.71 10^−3^ mmol), 31 μL etanol 16.5 μL of a solution of SDS in water (2.9 10^−4^ mmol) and 1 mL of citrate capped Au NPs aqueous solution under stirring. After 20 min N_2_ purge, polymerization was initiated by heated up to 70 °C, followed by addition of 100 μL of APS solution (0.02 M). The amount of AcSEO_2_MA ligand, ethanol concentration, and the appropriate reactive sequence addition, experimentally adjusted, were critical for the reaction success (Supplementary Fig. S1). After above 10 min, the solution became cloudy, indicating that polymerization started, and the solution was left to react for 2 h. To stop the reaction, the solution was cooling down in an ice bath while the tube was opened to air. Finally, after water dilution, the sample was four times centrifuged (4600 rpm, 30 min.) and resuspended in water until complete removal of empty polymer-particles and excess of reactants.

**Table 1:** Summary of Surfactant Concentration (SDS), Z Average Diameter and Polydispersity (PDI) and Zeta Potential (ζ) by DLS, Size Diameter by TEM (±Standard Deviation) and Surface Plasmon Band Wavelength (λ LSPR_max_) of Hybrid Nanoparticles with Different Polymer Shells.

Entry	Sample	SDS (M)	Water/Etanol	Z average^a)^ (nm)	PDI	ζ^a)^ (mV)	TEM (nm)	λ LSPR^a)^_max_ (nm)
Au	Au@citrate			64	0.13	−54.5	58 ± 6	543
G1	Au@pMEO_2_MA-G1	2.9 10^−4^	1/0.065	102	0.08	−44.7	91 ± 8	556
G2	Au@pMEO_2_MA-G2	1.4 10^−4^		177	0.02	−48.5	167 ± 11	565
G3	Au@pMEO_2_MA-G3	0.7 10^−4^		199	0.04	−40.4	190 ± 18	566
G4	Au@pNIPAM-G4	1.4 10^−4^	1/0.065	141	0.01	−34.6	198 ± 19	559
G5	Au@pNIPAM-G5	0.7 10^−4^		197	0.05	−35.6	307 ± 26	566
G6	Au@pMMA-G6	0.35 10^−4^	1/0.065	255	0.07	−57.6	262 ± 10	579
G7	Au@pMMA-G7	0.35 10^−4^	1/0.168	216	0.04	−55.0	223 ± 18	576
G8	Au@pSt-G8	0.35 10^−4^	1/0.065	71	0.15	−50.2	80 ± 11	549
G9	Au@pSt-G9	0.35 10^−4^	1/0.168	102	0.13	−49.5	120 ± 14	554, 689^b)^
G10	Au@pSt-G10	0.35 10^−4^	1/0.235	151	0.05	−52.53	166 ± 11	565
G11	Au@pSt-G11	0.35 10^−4^	1/0.300	198	0.04	−58.28	363 ± 27	567, 714^b)^

^a)^Z-average, ζ and λ LSPR_max_ for Au@pMEO_2_MA and Au@pNIPAM systems were determined at T 10 °C above the VPTT. ^b)^λ LSPR_max_ for Au@pSt shoulder.

### Characterization

Optical characterization was carried out in a Cary 3 BIO-Varian UV-Visible spectrophotometer equipped with a Peltier temperature control device. Transmission electron microscopy (TEM) images were recorded with a field emission scanning electron microscope (FESEM) Hitachi SU-8000 operated at 30 kV in transmitted electron imaging mode (S-TEM). Scanning electron microscopy (SEM) images were captured on a Hitachi SU-8000 at an accelerating voltage of 2.5 kV. A TEM and SEM grid was allowed to sit atop a drop of Au-nanohybrid solution for 2 h at 4 °C, while the atmosphere was being controlled to avoid drying of the solution. Hydrodynamic diameters (Z average) and Zeta potential (ζ) were measured by dynamic light scattering (DLS) using a Zetasizer Nano ZS instrument (Malvern Instruments Ltd, UK) equipped with a 4 mW He–Ne laser operating at a light source wavelength of 632 nm and a fixed scattering angle of 173° for detection. Malvern Dispersion Software was used for data acquisition and analysis, applying the general purpose algorithm for calculating the size distribution.

## Results and Discussion

In brief, as-synthesized citrate-stabilized AuNPs (d = 58 ± 6 nm)^7,29^ were centrifuged to remove citrate and the aqueous supernatant before they were added to a solution of ethanol/H_2_O containing acrylic- and vinyl-monomer, AcSEO_2_MA ligand and surfactant SDS. The one-pot synthetic method was achieved by final dropwise addition of APS to initiate the free radical polymerization of acrylic/vinyl monomer at 70 °C for 2 h.

**Figure 2:**
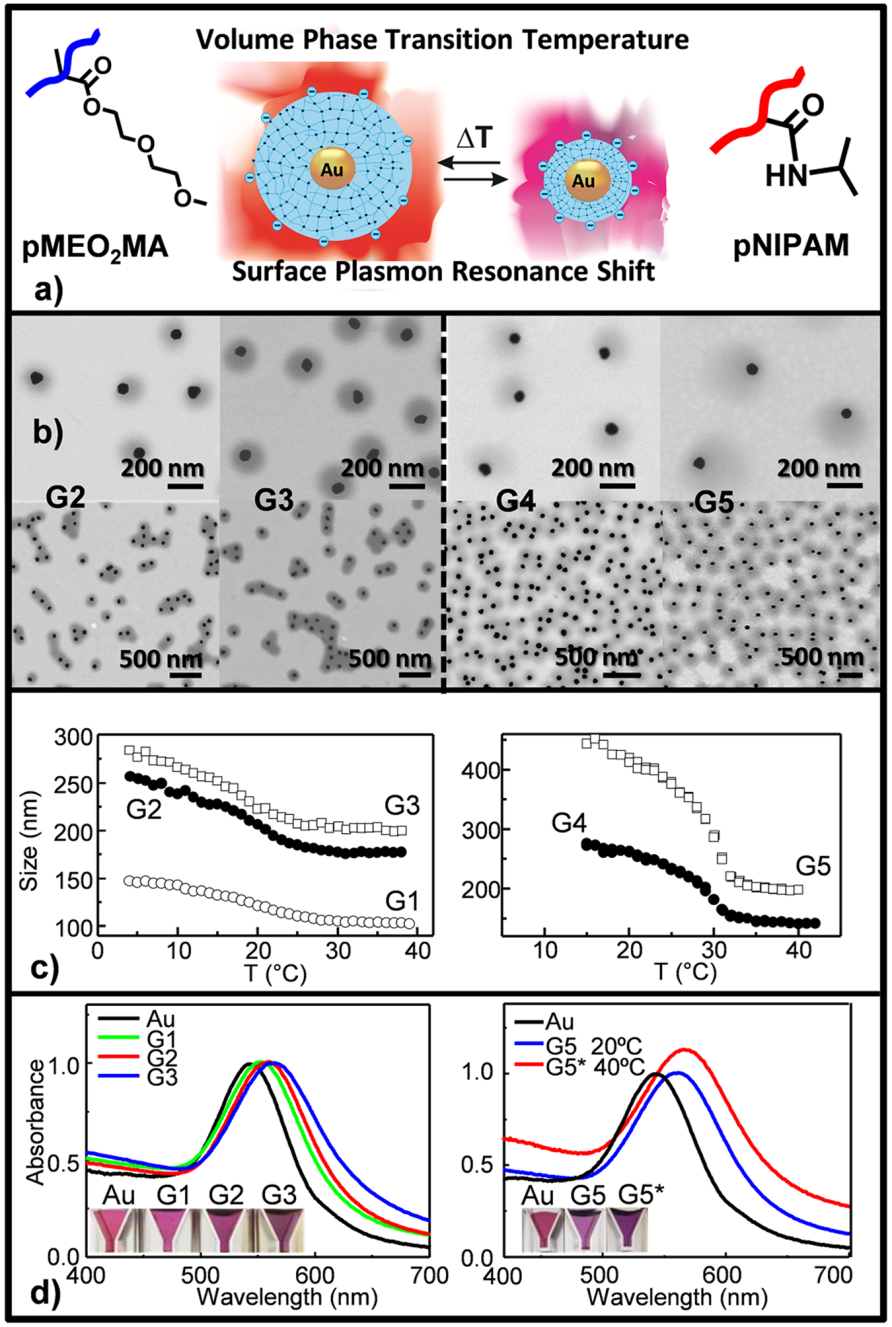
(**a**) Schematic illustrating temperature-induced swelling-shrinking behavior of AuNP encapsulated with pNIPAM (right) and pMEO_2_MA (left); (**b**) S-TEM images at high and low magnification and (**c**) Temperature-dependent hydrodynamic diameter evolution of AuNP@pNIPAM (right) and Au@pMEO_2_MA (left) with SDS concentration; and (**d**) UV-Vis spectra evolution of AuNPs with increasing pMEO_2_MA (left) shell thickness below VPTT, and with pNIPAM (right) encapsulation, at temperatures below (20 °C) and above (40 °C) the VPTT, for G5 sample. Insets: color variation of Au@pMEO_2_MA (left) and Au@pNIPAM (right) nanohybrid solutions.

Owing to their distinct hydrophobicity character, four acrylic/vinyl monomers, namely N-isopropylacrylamide (NIPAM), diethylene glycol methyl ether methacrylate (MEO_2_MA), methyl methacrylate (MMA) and styrene (St) (Fig. 1) were chosen as model for the AuNP system to parse the viability and versatility of our one-pot synthetic protocol. As first validation of the method, MEO_2_MA and NIPAM monomer, and AuNPs were investigated (Fig. 2a). pMEO_2_MA and pNIPAM are well known smart temperature-responsive polymers in water^30,31^, with improved swelling performances, once crosslinked. They were initially used to explore the optimal reaction conditions too (Table 1). Au@p(MEO_2_MA)-G1 sample in Table 1 indicates the initial experimental conditions on the basis of previous work^7^.

TEM images (Fig. 2b) shows *quasi* concentric core-shell morphology of the resulting Au@pMEO_2_MA (left-hand) and Au@pNIPAM (right-hand) hybrid NPs, with single Au-core inclusion per particle and anticipated increase metal surface coverage as SDS concentration decreases, independently of the polymer chemical-composition (Table 1, Fig. 2a). As can be seen, each nanohybrid displays almost uniform shell-thickness around the AuNP core (Fig. 2b), consistent with the polymer chain growing process. In Fig. 2, it can be observed that not all the cores are clearly in the NP centre. Note that even when NP hybrids would be completely concentric in solution; the interaction of the soft polymer-shell with the substrate and the dewetting process could result into the morphology shown in S-TEM images. In this scenario, the appropriate use of hydrophobic acetylthiolated methacrylic monomer ligand (AcSEO_2_MA) endows AuNP with a reduced interfacial energy and a fully amenable NP-surface, facing to monomer, which leads to multiple nucleation sites and eventually emerged shell. The presence of surfactant SDS stabilizes the NP accommodation inside the droplet as its hydrophobic-alkyl-arms interact within the outer-facing layer of the hydrophobic tagged-NPs. And, the feeble solubility of the pNIPAM and pMEO_2_MA in water at 70 °C, temperature above the lower critical solution temperature (LCST), leads to the growing polymer-shell collapse around the existing hydrophobic NP, which serves as nuclei for further networking-assembly. ζ-potential values collected in Tables 1 and 2 confirmed the colloidal stability of nanohybrids due to the negative charge at the particle surface. Indeed, it is recognized that the incorporation of initiator groups (anionic persulfate) into polymer chains introduces negative charges into nanogel particles^7,31^. Nevertheless, small contribution from residual SDS cannot be discarded, despite purification by two centrifugation-redispersion cycles of the samples. Table 2 also shows ζ-potential absolute value augmentation above VPTT. It is well established that thermo-responsive micro/nano particles, as synthesized by using persulfate initiator, exhibit charge density increase due to the volume contraction above the VPTT. In fact, this charge increase avoids particle aggregation after heating^31^.

**Table 2:** Z Average Diameter and Zeta Potential (ζ) at Temperature Below and Above the VPTT by DLS, Volume Temperature induced Phase Transition (VPTT), Swelling Ratio (*Q*) and Surface Plasmon Band Wavelength (λ LSPR_max_) of Au@polymer Hybrid Thermoresponsive Nanogels at Temperature Below and Above the VPTT.

Entry	Sample	Z average (nm)		ζ (mV)		VPTT (°C)	*Q*	λ LSPR_max_, (nm)	
		T < VPTT	T > VPTT	T < VPTT	T > VPTT			T < VPTT	T > VPTT
G1	Au@pMEO_2_MA-G1	149	102	−28.7	−44.7	18.2	3.0	554	556
G2	Au@pMEO_2_MA-G2	256	177	−25.6	−48.5	17.9	3.1	561	565
G3	Au@pMEO_2_MA-G3	282	199	−29.9	−40.4	21.3	2.9	563	566
G4	Au@pNIPAM-G4	276	141	−14.4	−34.6	29.6	7.9	556	559
G5	Au@pNIPAM-G5	444	197	−13.6	−35.6	30.3	11.6	559	566

For the two nanohybrid systems (Fig. 2a), the behavior brought by the core-shell morphology results from the main role played by the nanogel periphery in the temperature-induced volume phase transition (VPTT), which is accompanied by changes in hydrodynamic radius with temperature (Table 1, Fig. 2c and Supplementary Fig. S2). This thermo-response was assessed by dynamic light scattering

(DLS in DI water, Fig. 2c) and was confirmed by localized surface plasmon resonance (LSPR) analysis (UV-Vis, Fig. 2d). Additionally, the swelling behavior of our systems (Table 1), defined as the ratio (*Q*) between the volume of the gel at the swollen state and that of the gel in the fully collapsed state seemed to be influenced by the chemical details of the nanohybrid gel-shell. On the basis of this observation (Fig. 2c and Table 1), we believe that the higher hydrophilicity of NIPAM units together with their more flexible backbone, would favor the improved swelling capacity of pNIPAM nanogels compared to that of pMEO_2_MA nanogels, as it has been previously reported^32,33^.

Consistent with previous studies^7,34,35^, after polymer-shell encapsulation, the AuNP transverse plasmon-band (LSPR) remarkably red-shifted from that of the bare AuNPs (543 nm, Table 2 and Fig. 2d), indicating an increase in the refractive index near Au. Moreover, the absorption band of the Au@polymer nanohybrid was observed to shift to a longer wavelength at elevated temperatures (above LCST), which can be attributed to the shrinkage of the polymer-shell layer and the consequent increase in the refractive index of that layer^7,34–37^. This phenomenon could be observed directly by the naked eye (see the insets of Fig. 2d). The snapshots (Fig. 2d-insets) illustrate a color variation with thickness and temperature rise, induced by the particle scattering augmentation by size. Therefore, these results emphasize that, in our systems (Fig. 2 or Table 2), all NPs are singly embedded and well separated among them by polymer-shell gels; with no evidence of ¨plasmon coupling between adjacent-AuNPs¨-contribution to the associated refractive index increase around the AuNPs, once polymer collapse.

**Figure 3:**
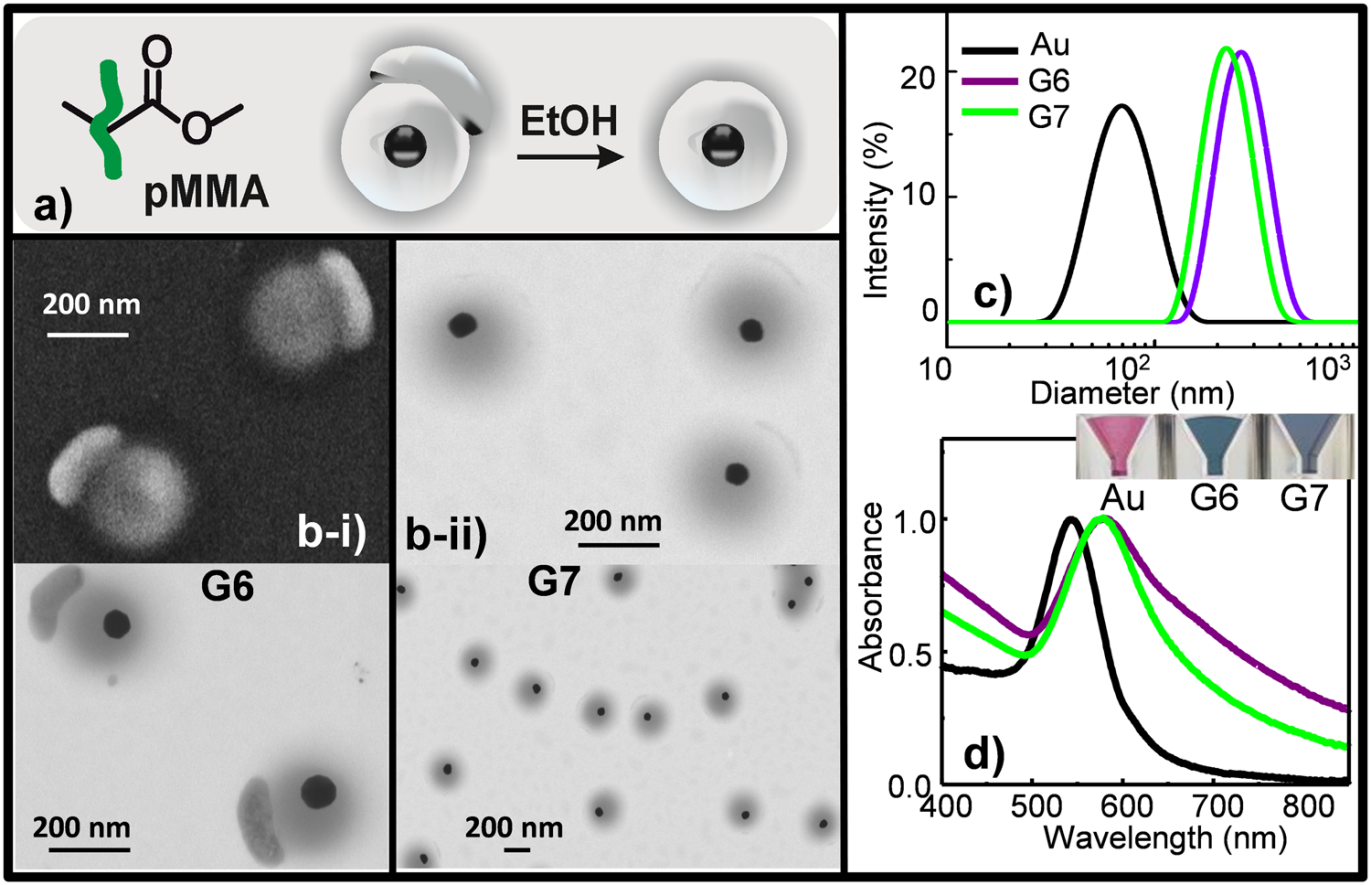
(**a**) Scheme of the monomer mediated *in situ* growth strategy for the core-shell Au@pMMA nanohybrids synthesis; (**b**) S-TEM images of nanohybrids obtained in different H_2_O/EtOH concentration ratio: (**b**-**i**) G6 and (**b**-**ii**) G7, both at high and low magnification. SEM image of G6 is reproduced in left-top of (**b**-**i**); (**c**) Size diameter by intensity distribution and (**d**) UV-Vis absorption of the nanostructures. Inset: color variation of the Au@pMMA nanohybrid solutions with the shell-morphology changes.

**Figure 4:**
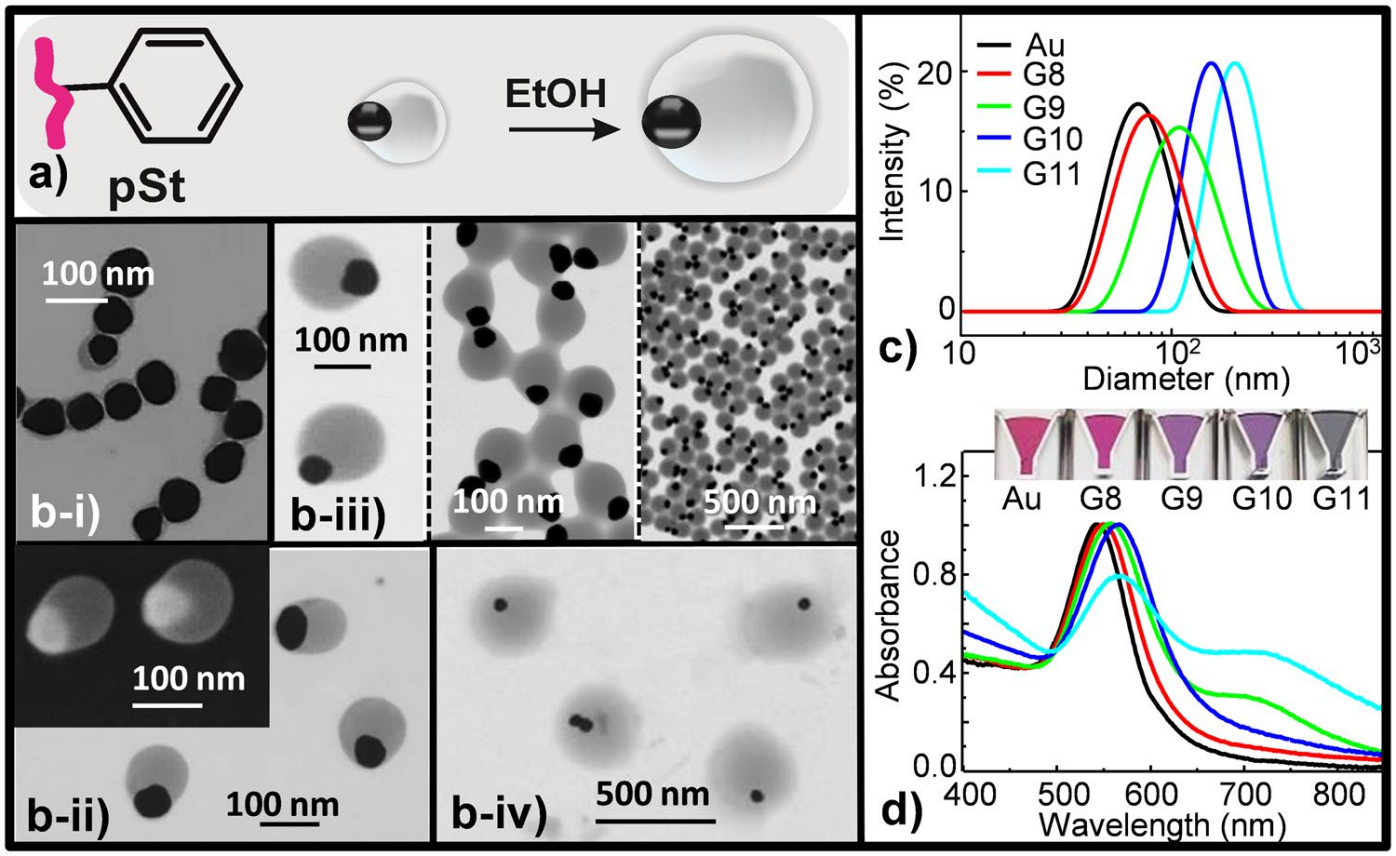
(**a**) Schematic depiction of eccentric or Janus core-shell nanohybrids consisting of pSt shell coverage anisotropically grown on AuNP; (**b**) S-TEM images of distinct morphologies of Au@pSt nanohybrids obtained with ethanol concentration increasing: (**b**-i) G8, (**b**-ii) G9, (**b**-iii) G10 at different magnification, and (**b**-iv) G11. Right-hand of (**b**-ii) illustrates SEM image of G9; (**c**) Size diameter by intensity distribution and (**d**) Extinction spectra of AuNP and the corresponding G8-G11 nanohybrids shown in b (i-iv). Inset: color variation of the Au@pSt nanohybrid solutions with shell-morphology evolution from concentric core-shell to eccentric and Janus-NPs.

To further demonstrate the adaptability and robustness of the method, the somewhat hydrophilic monomers (MEO_2_MA and NIPAM) were swapped for other more hydrophobic ones (MMA, Fig. 3a and St, Fig. 4a). Preliminary experiments with similar SDS concentration and water/ethanol ratio, as used for MEO_2_MA and NIPAM, resulted in lower pSt shell growth onto AuNP (data shown in Supplementary Fig S3). Note that pNIPAM and pMEO_2_MA, as water containing polymers, have swelling capability, not exhibited by pSt and pMMA. Then, since SDS controls nanohybrid size, at similar SDS concentration the nanohybrid´s polymer coverage for pNIPAM and pMEO_2_MA will be higher than for pSt and pMMA. In an attempt to overcome this limitation and to forge a broad-based coverage of the polymer, a lowered surfactant SDS concentration was added to the reaction mixture (Table 1) during the synthetic procedure (Fig. 1). The red-shift in extinction spectra (up to 579 and 549 nm, Au@pMMA-G6-Fig. 3d and Au@pSt-G8-Fig. 4d, respectively), due to the increase of the local dielectric constant surrounding the metal surface; and the particle size increase around 34%, Au@pSt-G8 and 300%, Au@pMMA-G6 (DLS, Table 1 and Fig. 3c) were consistent with polymer-shell formation around the Au core.

Surprisingly, when MMA was used instead of St, keeping constant all the other reaction parameters, significant differences were observed in the size and morphology of the formed nanohybrid. Representative TEM images showed anisotropic polymer-shell coverage of Au@pMMA-G6 (Fig. 3b-i) instead of the anticipated isotropic coverage attained when more hydrophilic polymers were used (Fig. 2b). Consistently with DLS data (Table 1), final polymer-coverage domain size seemed to be very affected by the conditions. Au@pMMA-G6 (Fig. 3b-i) exhibited a thick shell coverage (~10× times higher size augmentation); whereas thin polymer shell, with incipient Janus morphology, was achieved for Au@pSt-G8 (Fig. 4b-i). SEM image (Fig. 3b-i, left-top) supported the substantial irregularity, in appearance, of Au@pMMA-G6.

These results suggest a different nucleation/growth process arising from the two monomer hydrophobicities, as well as a dynamic barrier coverage onto AuNP surface, weak enough to permit the nuclei-growth process but strong enough to prevent agglomeration, in the regime of reduced surfactant concentration used (Table 1). Furthermore, the important differences in coverage increased size (about 8× times higher for Au@pMMA-G6 compared to Au@pSt-G8 together with the obtained morphology irregularities [Au@pMMA-G6] stressed the need to narrow down the key conditions for the improved coverage, in terms of size and morphology.

On the basis of evidence presented above, as well as more detailed results below, ethanol appears to drive the polymer-shell assembly of hydrophobic monomer on metal NPs, as induced by the slow addition of a good solvent to the monomer solution in water. Basically, the ethanol controls the polymerization of pMMA shells onto Au spheres (Fig. 3b, Table 1). Thus, the floating MMA phase acts as monomer source for the pMMA shell formation (Fig. 3b-i); and, indeed, the shell thickness continues a uniform growth, until the whole MMA amount is consumed (that eventually ends up engulfing AuNPs, Fig. 3b-ii). Nevertheless, this will only happen if the interfacial energies between the MMA droplets and aqueous phases, and surface ligands are quantitatively adjusted by the solvent (Fig. 3b-ii), at the initial stages of polymerization. Note that, from the point of view of interfacial energies, a key issue is to exchange the surface ligands on the core NPs, so that the subsequently formed shell can form a wetting layer around the core.

From the TEM images (Fig. 3b-i and ii), it can be clearly seen that the homogeneous nucleation (concentric shell) is promoted over the heterogeneous one (eccentric shell). So, whereas some AuNPs appeared not isotropically covered (Fig. 3b-i); nevertheless, AuNPs resulted uniformly enclosed for growing polymer shells (Fig. 3b-ii) by the addition of 2.6× times of ethanol. As previously mentioned, these morphologies might represent ¨late or ineffective¨ interfacial collisions/interactions in the residence ethanol volume ratio without ability for spreading of the monomer. The MMA monomer appears not to wet the AuNP completely, and, after the assembly, ethanol removal from the swollen polymer domain produces quizzical-aspheric pMMA shells by locking the resulting nanostructure (from hereafter emoji-N-hybrids, Fig. 3b-i). In fact, dense AuNP core inside swollen-like pMMA shell and compact pMMA, which ends forming a bump at the polymer-shell periphery, can be easily observed. Topological features in dark-field SEM image and electronic densities in the transmission electron beams in TEM picture reflect the distinct structural arrangements (porosities) of the components (Fig. 3b-i).

Upon the formation of pMMA-based nanohybrids, the surface plasmon resonance (SPR) peaks shifted from 543 nm (raw AuNP) to 576–579 nm (transverse plasmonic mode of Au@pMMA-G7 and Au@pMMA-G6 hybrids, respectively) and broadened with increasing shell thickness (Fig. 3d, Table 1). For emoji-N-hybrids (Au@pMMA-G6, Table 1), however, the SPR split into a second absorption peak, the longitudinal plasmonic mode (weak shoulder at 670 nm), suggesting the formation of irregular polymer shell on metallic nanostructures. These wavelengths result from the changes of refractive index from the inhomogeneous polymer reorganization around the Au-cores. Interestingly, the different adaptation of pMMA chains to variations in the medium condition (solvent/no-solvent ratio), involve the formation of two well differentiated molecular-structure arrangements of the polymer (a denser pMMA protuberance and a lighter pMMA shell-coverage) around the gold-core, which determine in last term the optical response of the metal NP attributable to the local refractive index contrasts of its surroundings (solvent, polymer). It is important to notice that the refractive index of pMMA protuberance generates additional broadening of the plasmonic band due to the damping of the SPR in AuNPs (purple line in Fig. 3d). On the other hand, no evidence of ¨plasmonic coupling of adjacent Au-cores¨ contribution was found (Fig. 3b-ii). This plasmon band process is complemented with a color variation of the nanohybrid solution from dark red to greenish blue (inset of Fig. 3d).

Aware of the particular relevance of ethanol/water ratio in the nanohybrid generation, in terms of their morphologies and successful shell-growth process, we programmed a set of experiments to evaluate the role of ethanol as co-solvent in forming the pSt-based hybrid NPs, keeping all the other synthetic factors constant (Fig. 4, Table 1). A mere glance to TEM pictures (Fig. 4b) and DLS data (Fig. 4c) revealed different degrees and shapes of polymer surface coverage. With the increase of ethanol contribution the morphology transited from eccentric core-shell or Janus NPs (Fig. 4b-i-iii) to more concentric core-shell NPs (Fig. 4b-iv) in selective solvent (ethanol/water) ratios. Actually, 2.6× , 3.6× and 4.6× times of ethanol addition involved controllable surface coverage increase of about 44%, 111% and 180% respectively (Z average in Table 1). In the range of the Janus-type nanostructure (Au@pSt-G9), a further rise of ethanol contribution leads to a less defined periphery of pSt coverage, and facilitates an ordered hexagonal pattern distribution of hybrids on substrates, likely due to electrostatic repulsion during drying process. This may be due to the ethanol ability to swell the pSt chains, which permits boundary defects (paths) creation on the surface-coverage as the solvent is expelled from the polymer domain, together with the pSt chains move outside during the synthesis process. These paths can easily connect each other under conditions of high hybrid NPs concentration. This effect can be observed in Fig. 4b-iii. SEM (Au@pSt-G9, right-hand of Fig. 4b-ii) image clearly illustrate the Janus-type nanohybrid structure with a hard gold core anisotropically surrounded by a soft pSt polymer-shell, which gradually becomes coarser with the improved presence of ethanol (Table 1).

These observations so far can be explained through the nuclei-growth approach^16,17,19^, where the heterogeneous nucleation step assumes a crucial role in determining the final eccentric to Janus morphology of nanohybrids. During the pSt shell-assembly, the styrene vinyl-monomer, which solubility increases by ethanol, is adsorbed on the ligand tagged-NP surface, driven by van der Waals and hydrophobic interactions. Owing to the higher hydrophobicity of the St, the selective pSt adsorption is also determined by the partitioned surface functionalities (hydrophilic citrate and hydrophobic AcSEO_2_MA ligands) on AuNPs through competitive ligand coordination^38^. The increase in hydrophobicity would also create the interfacial tension with the hydrophilic AuNP surrounding; and the higher surface tension facilitates phase separation^39,40^, making such a surface prone to pSt deposition in an heterogeneous nucleation way. Thus, once the St monomer anchor to the AuNP surface start growing through continuous precipitation polymerization, leading to an eccentric and Janus-type structure (Fig. 4).

The morphological changes were found to affect the localized surface plasmon resonance (LSPR) of the resulting core-shell hybrid NPs. Note that, AuNPs are very sensitive to the refractive index of their surface vicinity^41^; and consequently, a sample with inhomogeneous coverage will lead to both redshift (from 543–567 nm) and broadening of the transverse plasmonic band (Fig. 4d).

Moreover, the peculiar ¨eccentric¨ morphology of the hybrids can favor the interparticle interaction between adjacent Au-domains, which can induce the appearance of the second absorption peak (longitudinal plasmonic band)^5^ (Fig. 4d). As mentioned before, the St hydrophobicity augmentation provokes phase separation and protrudes hydrophilic AuNPs beyond the pSt coverage (Fig. 4b)^16,17,42^. Then, the slight Au core relocation from the pSt coverage can trigger coupling effect from different projected Au faces^5^.

At this point, we might say that lower ethanol concentration leads to thinner polymer shell (Au@pSt-G9); whereas higher concentration disturbs the proper functioning of coverage-boundary, and subsequently, a broader and red-shifter longitudinal plasmon band is defined (Au@pSt-G11, Fig. 4). This boundary distortion enables rapprochement among well-defined hybrids (Supplementary Fig. S4), which can be then disassembled by simple addition of water (Fig. 4b-iv). Hence, the summation of the ¨plasmonic coupling¨ effect to the ¨surrounding refractive index¨ effect should be taken into consideration. And, both the shifted Au absorption peak and the appearance of additional peak in longer wavelength range well support the symmetry breaking of the nanohybrids during the polymer shell growth.

## Conclusion

In summary, we report a simple and versatile ¨one-pot¨ radical polymerization strategy to obtain Au-polymer ¨core-shell¨ hybrid NPs with tunable polymer-coverage asymmetry, regardless of the monomer polarity. The strategy relies on the use of thiolated methacrylate-monomers as compatible bridge between the as-synthesized citrate-capped Au-surface and the growing polymer-chains, based on the high affinity of thiol group by the gold surface. To demonstrate our concept, four monomers of increasing hydrophobicity NIPAM < MEO_2_MA < MMA < St have been selected. Most importantly, the successful controlled polymer-growth onto outside Au-periphery is found to dramatically depend on the fine balance between AuNPs and thiolated-monomer; whereas increasing monomer hydrophobicity requires an additional tuning of the ethanol/water ratio. Moreover, the environment responsive transformation of AuNPs and the control of interfacial energy among components provide singular nanohybrid species, assembled into concentric plasmonic NPs-core and thermo-responsive polymer-shells or eccentric core-shell structures (Janus-type and Emoji-N hybrids). Actually, new asymmetric ¨Emoji-N hybrid¨ nanostructures, and Janus-type nanohybrids were built-up by decreasing monomer polarity in the mixed solvent of ethanol/water. This approach offers well control over nanohybrid morphologies, thus fine-tuning the optical properties (LSPR position and shape modulation) of assembled Au-polymer nanostructures, opening up a broader applicability in fields as biomedical, optoelectronic, photonic and catalysis.

## Electronic supplementary material

Supplementary Information
