# Supplementary material for: Synthesis of Au@polymer nanohybrids with transited core-shell morphology from concentric to eccentric Emoji-N or Janus nanoparticles
Source: Sci Rep. 2018 Apr 10;8:5721. doi: 10.1038/s41598-018-24078-8 (PMC5893630; doi:10.1038/s41598-018-24078-8)
Supplement: Supplementary file 1 — Supplementary Information [file 41598_2018_24078_MOESM1_ESM.pdf]

Supplementary Information

Synthesis of Au@polymer nanohybrids with transited core-shell morphology from concentric to eccentric Emoji-N or Janus nanoparticles

Nekane Guarrotxena, Olga García and Isabel Quijada-Garrido**

Group of Nanohybrids and Interactive Polymers (NyPI), Instituto de Ciencia y Tecnología de Polímeros. Consejo Superior de Investigaciones Científicas (ICTP-CSIC), c/ Juan de la Cierva, 3. E-28006 Madrid, Spain.

N.G.: E-mail: nekane@ictp.csic.es; Tel: (+34) 915622900

I.Q-G.: E-mail: iquijada@ictp.csic.es; Tel: (+34) 915622900

List of Contents

Synthesis of 2-[2-(2-(acetylthio)ethoxy)ethoxy]ethyl methacrylate ($AcSEO_2MA$)…………………...S2

Synthesis of 2-(2-chloroetoxy)ethoxy)ethyl methacrylate…………………………………..…....S2

Cartoon depicting the synthetic route of $AcSEO_2MA$ (Scheme S1)……………………….……….....S2

Bright field S-TEM images corresponding to a preliminary synthesis of Au@pMEO_2MA without $AcSEO_2MA$ (Figure S1) ……………………………….…………………………….…….S3

DLS measurements for Au@pMEO_2MA (G2, G3) and Au@pNIPAM (G4, G5), at temperature below and above VPTT (Figure S2)……..……………….…………………………….…....S3

Bright field S-TEM image corresponding to a preliminary synthesis of Au@pSt nanohybrid (Figure S3)………………………………………………………………………….………S4

Bright field S-TEM image of Au@pSt-G11 nanohybrid (Figure S4)……………..……………..…...S4

Synthesis of 2-(2-(2-(acetylthio)ethoxy)ethoxy)ethyl methacrylate ($AcSEO_2MA$) (2): The monomer $AcSEO_2MA$ was synthesized according the two-step protocol described *Scheme S1*:

Synthesis of 2-(2-(2-chloroethoxy)ethoxy)ethyl methacrylate (1). Methacryloyl chloride (0.131 mol, 13.708 g) was added dropwise over a solution of 2-(2-(2-chloroethoxy)ethoxy)ethan-1-ol (0.087 mol, 14.742 g) in 150 mL of anhydrous dichloromethane in the presence of triethylamine (0.095 mol, 9.854 g) at 0 °C. Afterwards, reaction was allowed to reach room temperature and maintenance overnight in inert atmosphere. After, the solution was filtered to remove triethylamine chlorohydrate, washed with water (3 × 50 mL) and dried over anhydrous sodium sulfate. The resulting monomer was obtained as a yellow liquid. 18.77 g (yield 91%) and was used in the next step without purification (1). ^{1}H-RMN (400MHz, $CDCl_3$): δ (ppm) 6.10 (s, 1H, CHH=C(CH_3)-), 5.55 (s, 1H, CHH=C(CH_3)-), 4.28 (t, 2H, -CH_2OC=O), 3.73 (t, 2H, -CH_2Cl), 3.65-3.58 (m, 8H, -CH_2O-), 1.92 (s, 3H, CH_2=C(CH_3)-)

In a second step, the 2-(2-(2-chloroethoxy)ethoxy)ethyl methacrylate (1) (0.095 mol, 22.485 g) obtained, potassium thioacetate (0.105 mol, 12.016 g), and potassium iodide (0.105 mol, 12.016 g) were dissolved in 400 mL of acetonitrile. The solution was heated to reflux for 24 h at 80 °C. The crude was filtered, removed the acetonitrile and then extracted with ethyl acetate and washed with water. The organic was dried with anhydrous sodium sulfate and separated by column chromatography using as eluent a mixture of hexane/ethyl acetate (7:3 v/v). The resulting monomer (2) was obtained as an orange-yellowish liquid. 7.7 g de $AcSEO_2MA$ (yield 30%).^{1}H-RMN (400MHz, $CDCl_3$): δ (ppm) 6.13 (s, 1H, C*H*H=C(CH_3)-), 5.58 (s, 1H, C*H*H=C(CH_3)-), 4.30 (t, 2H,-CH_2OC=O), 3.75 (t, 2H, -SCH_2CH_2O-), 3.67-3.58 (m, 6H, -CH_2O-), 3.09 (t, 2H, -CH_2S-), 2.33 (s, 3H, CH_3C=O), 1.95 (s, 3H, CH_2=C(CH_3)-).

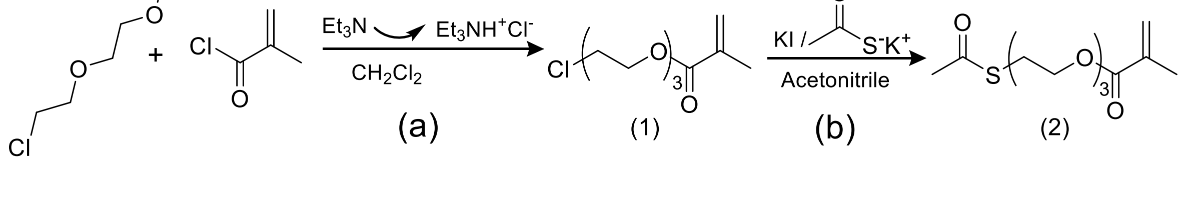

Scheme S1. Reaction to obtain (a) 2-(2-(2-chloroethoxy)ethoxy)ethyl methacrylate and (b) 2-(2-(2-(acetylthio)ethoxy)ethoxy)ethyl methacrylate ($AcSEO_2MA$).

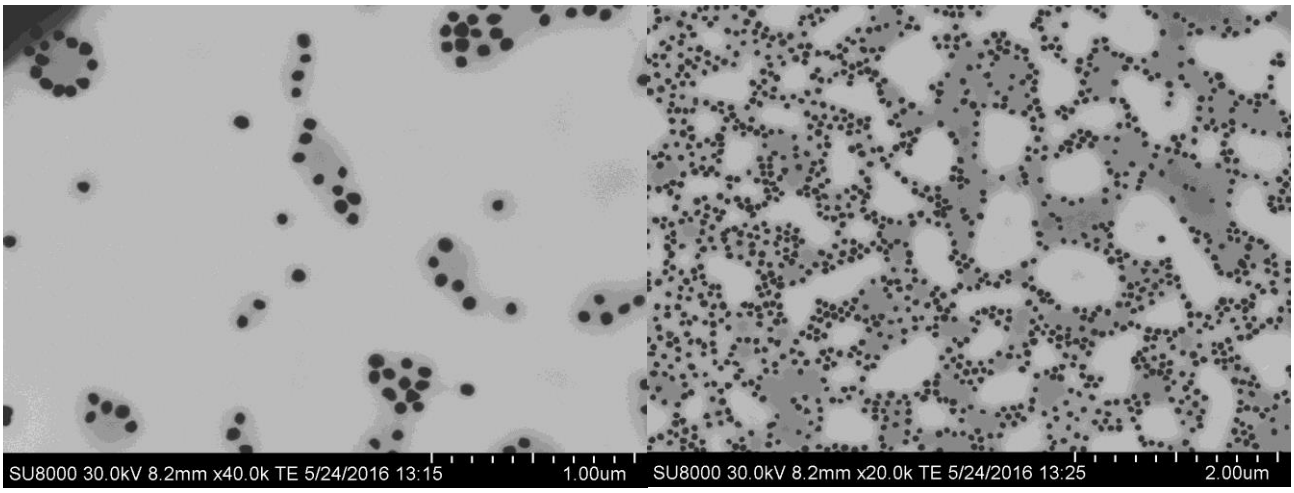

Figure S1. Bright field S-TEM images corresponding to a preliminary synthesis of Au@pMEO$_2$MA nanohybrid synthesized with the same experimental conditions as sample G2 but without AcSEO$_2$MA monomer ligand. They show unsuccessful synthetic results which evidence the critical role of AcSEO$_2$MA.

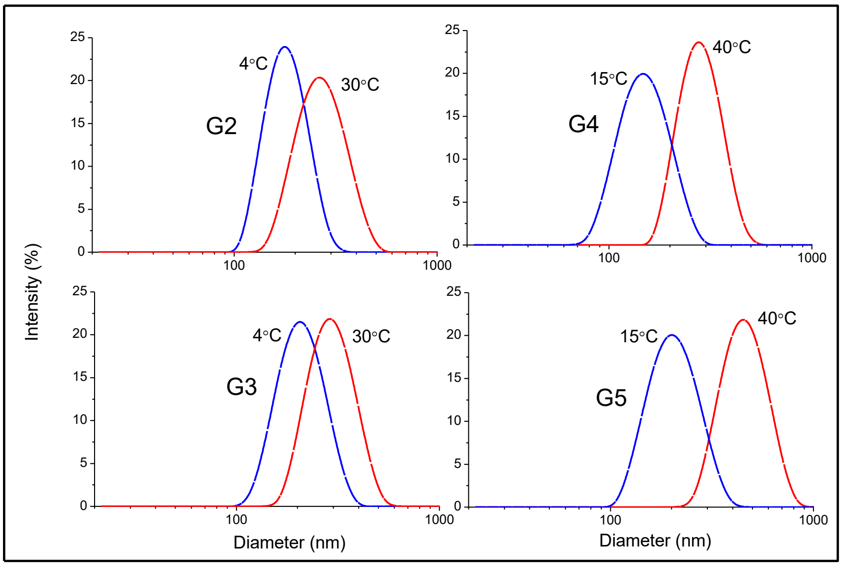

Figure S2. Size diameter by intensity as determined by DLS for samples G2 and G3 (Au@pMEO$_2$MA) and G4 and G5 (Au@pNIPAM), at temperature below and above VPTT (blue and red curves, respectively).

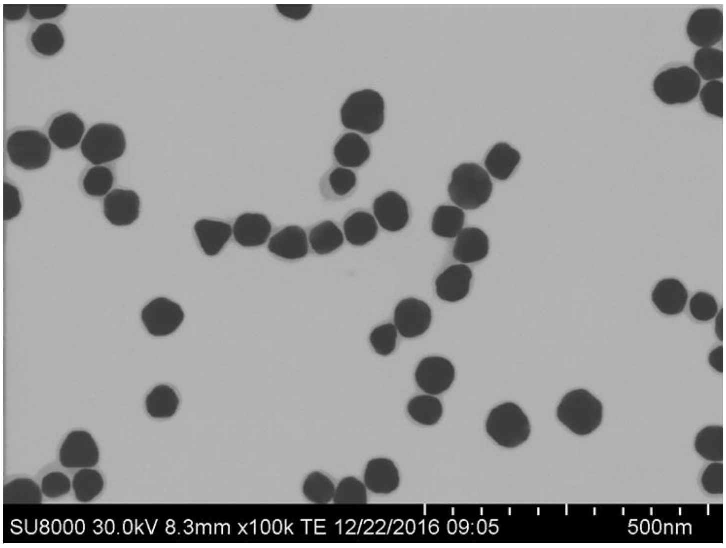

Figure S3. Bright field S-TEM image corresponding to a preliminary synthesis of Au@pSt nanohybrid synthesized with a concentration of SDS of 0.7 10^{-4} M and a water/ethanol ratio of 1/0.065.

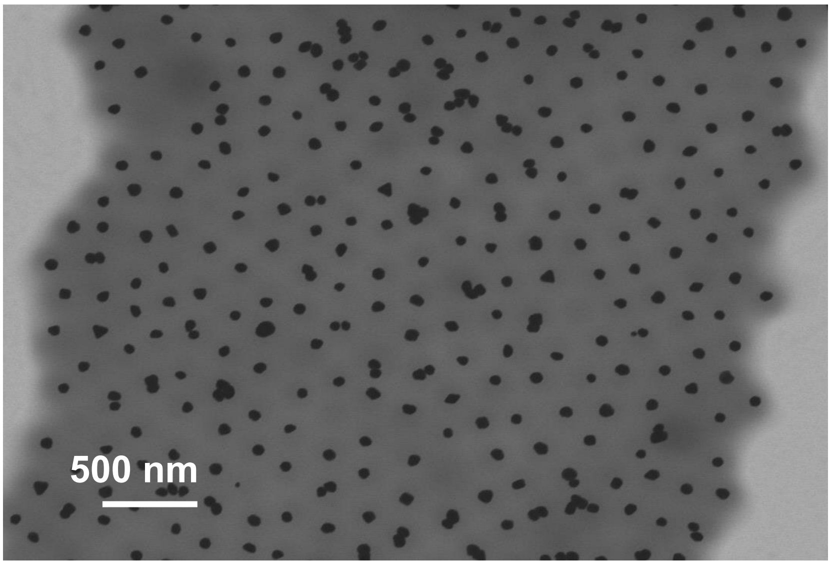

Figure S4. Representative bright field S-TEM image of Au@pSt-G11 nanohybrid.
